# Transcriptional dynamics of *Chitinophaga* sp. strain R-73072-mediated alkannin/shikonin biosynthesis in *Lithospermum officinale*

**DOI:** 10.3389/fmicb.2022.978021

**Published:** 2022-08-22

**Authors:** Muhammad Ahmad, Alicia Varela Alonso, Antigoni E. Koletti, Andreana N. Assimopoulou, Stéphane Declerck, Carolin Schneider, Eva M. Molin

**Affiliations:** ^1^Center for Health & Bioresources, AIT Austrian Institute of Technology GmbH, Tulln, Austria; ^2^Department of Botany and Biodiversity Research, University of Vienna, Vienna, Austria; ^3^Institut für Pflanzenkultur GmbH & Co. KG., Schnega, Germany; ^4^Earth and Life Institute, Mycology, Université catholique de Louvain, Louvain-la-Neuve, Belgium; ^5^School of Chemical Engineering, Laboratory of Organic Chemistry and Center for Interdisciplinary Research and Innovation of AUTh, Natural Products, Research Centre of Excellence (NatPro-AUTh), Aristotle University of Thessaloniki, Thessaloniki, Greece

**Keywords:** alkannin/shikonin, *Chitinophaga*, plant defense, transcriptome, jasmonate, ethylene, salicylic acid

## Abstract

Plants are colonized by a wide range of bacteria, several of which are known to confer benefits to their hosts such as enhancing plant growth and the biosynthesis of secondary metabolites (SMs). Recently, it has been shown that *Chitinophaga* sp. strain R-73072 enhances the production of alkannin/shikonin, SMs of pharmaceutical and ecological importance. However, the mechanisms by which this bacterial strain increases these SMs in plants are not yet understood. To gain insight into these mechanisms, we analyzed the molecular responses of *Lithospermum officinale*, an alkannin/shikonin producing member of Boraginaceae, to inoculation with R-73072 in a gnotobiotic system using comparative transcriptomics and targeted metabolite profiling of root samples. We found that R-73072 modulated the expression of 1,328 genes, of which the majority appeared to be involved in plant defense and SMs biosynthesis including alkannin/shikonin derivatives. Importantly, bacterial inoculation induced the expression of genes that predominately participate in jasmonate and ethylene biosynthesis and signaling, suggesting an important role of these phytohormones in R-73072-mediated alkannin/shikonin biosynthesis. A detached leaf bioassay further showed that R-73072 confers systemic protection against *Botrytis cinerea.* Finally, R-73072-mediated coregulation of genes involved in plant defense and the enhanced production of alkannin/shikonin esters further suggest that these SMs could be important components of the plant defense machinery in alkannin/shikonin producing species.

## Introduction

In nature, plants are associated with a wide range of microorganisms such as bacteria, fungi, and archaea, many of which stimulate important plant functions related to growth and health (Compant et al., 2019; Song et al., 2021; Thoms et al., 2021). Several bacterial species belonging to the genera *Pseudomonas, Burkholderia*, *Acetobacter*, or *Azospirillum,* among others, have been demonstrated to stimulate growth, enhance tolerance to abiotic stresses, and activate systemic resistance against a variety of pests and diseases in different plant species (Kumar and Verma, 2018; Mhlongo et al., 2018). These beneficial bacteria trigger systemic resistance, where the colonized hosts deploy rapid cellular defense responses including secondary metabolites (SMs) biosynthesis upon subsequent infection by pathogens (Conrath et al., 2006; Kumar and Verma, 2018; Mhlongo et al., 2018; Yu et al., 2022). For example, inoculation of *Arabidopsis thaliana* with *Pseudomonas fluorescens* SS101 and *Sphingomonas melonis* Fr1 led to improved production of defense-related camalexin and/or glucosinolate SMs (van de Mortel et al., 2012; Ryffel et al., 2016; Vogel et al., 2016). In non-model plants, such as medicinal herbs, endophytic microorganisms (microorganisms that colonize the host internally; Hardoim et al., 2015) have been shown to induce pharmaceutically important SMs improving their therapeutic properties (Köberl et al., 2013; Huang et al., 2018). For instance, Ray et al. (2019) showed that an endophytic bacterium of the genus *Acinetobacter* increased morphine and thebaine contents of *Papaver somniferum*. Furthermore, the colonization of *Echinacea purpurea* by endophytic bacteria modulated the biosynthesis of immunomodulator alkamides (Maggini et al., 2017).

Alkannin, its enantiomer shikonin, and its plethora of derivatives (A/S) are pharmaceutically bioactive SMs mainly produced in the root periderm of Boraginaceae (Yazaki, 2017), and the A/S biosynthesis pathway has been already extensively studied in different Boraginaceae species including *Lithospermum officinale* (Rai et al., 2018; Ahmad et al., 2022), *L. erythrorhizon* (Yazaki, 2017; Takanashi et al., 2019; Auber et al., 2020; Song et al., 2020, 2021; Suttiyut et al., 2022), *Echium plantagineum* (Tang et al., 2020), and *Arnebia euchroma* (Wang et al., 2019). Because of their interesting pharmaceutical properties and significant commercial value (Papageorgiou et al., 1999, 2008), there has been increasing interest in enhancing their production, e.g., through boosting its biosynthetic pathway, also by using beneficial microbes (Rat et al., 2021). Recently, Fazal et al. (2021) and Rat et al. (2021) demonstrated that A/S producing Boraginaceae (e.g., *Alkanna tinctoria*; Ahmad et al., 2021) host diverse microbial communities, among which a number of cultivable bacteria were shown to improve A/S production in a hairy root culture system. These findings were further supported by a study from Varela-Alonso et al. (2022), in which endophytic bacteria isolated from *A. tinctoria* also improved A/S biosynthesis in *L. officinale*, a Boraginaceae widely distributed in Europe and Asia (Al-Snafi, 2019). Among the tested bacterial isolates, *Chitinophaga* sp. strain R-73072 (herein referred as R-73072) showed the highest induction in total A/S biosynthesis (Rat et al., 2021). However, the molecular mechanisms involved in the specific R-73072–*L. officinale* interaction and how R-73072 improves A/S production in *L. officinale* remain fully unknown.

It is well established that beneficial microorganisms interfere with phytohormonal signaling where jasmonate (JA), salicylic acid (SA), and ethylene (ET) play critical roles in the signaling cascades during plant-microbe interaction regulating various biological processes including the induction of systemic resistance (Niu et al., 2011; Pieterse et al., 2014; Nie et al., 2017). For example, *Bacillus cereus* AR156 provided systemic protection to *Arabidopsis thaliana* against *Pseudomonas syringae pv. tomato* DC3000 by simultaneously activating SA, JA, and ET signaling pathways (Niu et al., 2011). Similarly, *Pseudomonas fluorescens* SS101 systemic protection in *Arabidopsis thaliana* was dependent on SA signaling and enhanced production of indolic glucosinolates (van de Mortel et al., 2012). In Boraginaceae, also the A/S biosynthesis is tightly regulated by phytohormones. JA or its methyl derivative (MeJA) and ET positively regulate A/S production (Yazaki, 2017), while SA either acts as a negative regulator (Kumar et al., 2014) or does not influence A/S biosynthesis (Yazaki et al., 1997a; Ahmad et al., 2022), depending on the cultivation system and plant species. Considering the positive role of JA and ET in A/S production and microbial interference with phytohormonal signaling, we hypothesize that R-73072 inoculation will result in strong induction of A/S production with concomitant activation of JA and/or ET biosynthesis, signaling, and defense-related genes in Boraginaceae. Since beneficial microorganisms generally protect host plants against a wide range of phytopathogens (Pieterse et al., 2014), we further expected that R-73072 inoculated plants will be protected against subsequent infection by the phytopathogen.

To test these hypotheses and to provide mechanistic insight into the microbe-plant interaction, we inoculated *L. officinale* with R-73072 in a gnotobiotic system and performed mRNA-seq and targeted metabolite profiling of inoculated and non-inoculated roots. In addition, we performed a bioassay by inoculating *L. officinale* roots with R-73072 and subsequently challenged the leaves with the fungal pathogen *Botrytis cinerea* to determine whether R-73072 provides systemic protection against this well-known plant pathogen.

## Materials and methods

### Bacterial and plant culture

*Lithospermum officinale in vitro* plants (clone 16) were provided by INoQ GmbH (Schnega, Germany) and grown and maintained on a modified Murashige and Skoog (MS^mod^) medium, as described in Varela-Alonso et al. (2022). A pure culture of *Chitinophaga* sp. strain R-73072 (Rat et al., 2021) was provided by BCCM/LMG Bacteria Collection (Ghent, Belgium). Bacterial inoculum was prepared as described in Varela-Alonso et al. (2022). Briefly, the bacterium was grown in a 35 ml liquid R2B medium (Supplementary Data S0A) at 25°C and 100 rpm for 72 h. A total of 5 ml was pipetted and transferred to a 15 ml tube for enumeration without affecting the rest of the culture. The falcon tube containing the 30 ml left was centrifuged at 4°C, 14,000 rpm for 10 min. The supernatant was discarded, and the pellet was resuspended in 2 ml of R2B medium supplemented with 10% glycerol and frozen at –20°C until use. Bacterial colony-forming units were enumerated by preparing dilutions from 10^−2^ to 10^−8^ and were then plated in triplicates on an R2A medium. Bacterial colonies were quantified after 1 week when bacterial growth became visible.

### Experimental design

For each treatment, 4–5 glass jars, each containing three individual plantlets, were maintained throughout the experiment. The glass jar containing the plantlets was maintained under long-day conditions (16:8, light: dark) with light intensity of 50 μmol m^−2^ s^−1^ and alternating day and night temperature (21–22°C/18–20°C). The bacterium was inoculated *in vitro* to the plants as follows: first, the inoculum was re-suspended in sterile phosphate-buffered saline (PBS) at pH 7.4 and adjusted to 10^6^ colony forming units (CFU). Bacterial solution of 10 μl was introduced inside the solid medium with a micropipette. For control plants, 10 μl of sterile PBS was introduced into the medium in a similar way as that for the bacterial treatment. Four-week-old plants were selected, and 2–3 cm long shoot cuttings were cut starting from the tip. The shoot cuttings were immediately transferred to the modified Strullu-Romand medium (MSR^mod^; Varela-Alonso et al., 2022) in contact with the bacterial or PBS suspension. Three shoot cuttings were transferred per glass jar. To avoid the possible inhibition of shikonin production by roots growing under light conditions, a 1 cm layer of sterile sand was added to the top of the medium surface. The jar periphery was covered with aluminum foil and taped. Root tissues were harvested after 5 weeks of bacterial treatment (~2–3 weeks after root emergence from shoot cuttings). For each replicate, the roots of three individuals from the same jar were washed lightly to remove sand and agar, pooled together, and immediately flash-frozen in liquid nitrogen. The frozen tissues were stored at –80°C for further analysis.

### *Botrytis cinerea* inhibition assay

Three-week-old plants of *L. officinale* were grown in MS^mod^ medium as described above and were transferred to the greenhouse in a twice sterilized (145°C for 10 h in an oven) potting substrate consisting of calcinated clay and quartz sand of two size categories (0.4–0.8 mm and 1–2 mm) in a proportion of 2:2:1 (v:v:v). Prior to transfer to the potting substrate in pots (3 cm diameter), the roots of individual plants were rinsed with sterilized water to remove agar medium and 10 plants with intact roots were inoculated with 1 ml (10^6^ CFU/ml in PBS buffer) of *Chitinophaga* sp. strain R-73072 culture. An additional set of 10 plants received only sterilized PBS. For inoculation, bacterial inoculum or PBS was dispensed over the rinsed roots and immediately covered with the potting substrate. After 4 weeks of acclimatization in the greenhouse under natural light and temperature (20–33°C), the plants were transferred to 1 l pots filled with the same substrate. At this time, a second inoculation was performed by dispensing by 1 ml (10^6^ CFU/ml) of bacterial culture in PBS into the root zone of the plant. Control plants were inoculated in a similar way with PBS. The plants were then grown for 2 weeks before the *Botrytis cinerea* inoculation. *Botrytis cinerea* strain DSM 4709 (DSMZ, Braunschweig) was routinely maintained on a ½-strength PDA medium for 21 days at 20°C under a 12 h photoperiod prior to infection. For the infection assay, one leaf of every plant was cut and placed in a 9 cm Petri plate with agar water, then a gel plug of 4 mm of a 3-week-old *Botrytis cinerea* was placed in the center of every leaf, in sterile conditions. The plates were maintained in a chamber at 20°C, under a 16-h light photoperiod for 3 days. A picture of every Petri plate was taken, and ImageJ software was used for measuring the diameter of the infected area.

### RNA extraction and qRT-PCR

Approximately 40 mg of frozen root tissues were pulverized in liquid nitrogen, and RNA was extracted using the RNeasy Plant Mini Kit (Qiagen, Hilden, Germany). Extractions were performed using the manufacturer’s instruction except that after adding RLT buffer, samples were heated at 56°C for 3 min. To avoid genomic contamination, a DNAse treatment was performed using RNase-Free DNase Set (Qiagen, Hilden, Germany) following the manufacturer’s protocol.

Expression of three genes (Leryth_015068: LePGT1, Leryth_016594: LeCYP76B101, and Leryth_014271: HMGR) of the A/S pathway was analyzed using qRT–PCR prior to mRNA sequencing. RNA extracts (1 μg each), that were later also used for mRNA-seq, were reverse transcribed using iScript Transcription Supermix (Bio-Rad, CA, United States) according to the manufacturer’s protocol. The cDNAs were diluted to 50 ng/μl, and 200 ng were used as a template in a mixture containing 1× SsoAdvanced Universal SYBR Green Supermix (Thermo Fisher Scientific) and 200 nM of forward and reverse gene-specific primers. For LePGT1 and HMGR, we used previously published primer sequences (Wu et al., 2009; Auber et al., 2020), while for LeCYP76B101, new gene-specific primers were designed (Supplementary Data S0B). The qRT–PCR reaction was performed using CFX Connect Real-Time PCR System (Bio-Rad, CA, United States) set up with the following conditions: 3 min at 95°C, 45 cycles of 15 s at 95°C, and 35 s at 58°C followed by melt curve analysis from 55°C to 95°C. The normalization of the target was based on transcript abundance of LeACT7 (Izuishi et al., 2020; Ueoka et al., 2020) as an internal standard and estimated using the 2^−ΔΔCt^ method (Livak and Schmittgen, 2001).

### mRNA sequencing and data analysis

Total RNA was submitted to the Next Generation Sequencing Facility of the Vienna BioCenter Core Facilities (VBCF) for library preparation and mRNA sequencing. After RNA quality control with Agilent’s Bioanalyzer, mRNA libraries were prepared using a polyA capture method (NEB, poly-A) and were sequenced as 150 bp paired-end on the Illumina NovaSeq S2 platform. For mRNA-seq, four replicates, where each replicate represents a pool of roots from three individuals growing in the same glass jar, were sequenced. Raw data are deposited in the NCBI sequence read archive under the BioProject number PRJNA854093.

Unless otherwise stated, default parameters were used for each analysis. The raw reads were quality controlled using FastQC v0.11.5 (Andrews, 2010) and were further trimmed for adapters and low-quality reads (*Q* < 20). Quality trimming was performed with BBDuk v37.68 (Bushnell, 2019) and high-quality reads of at least 50 bp length were mapped to the genome of *L. erythrorhizon* v1.0 (Auber et al., 2020) using HISAT2 v2.1.1 (Kim et al., 2019), followed by mapping quality assessment using Qualimap v2.2.1 (Okonechnikov et al., 2016). To generate read counts, featureCounts was used in paired-end and strand-specific mode, taking into account only uniquely mapped reads (Liao et al., 2014). For differential gene expression analysis, DESeq2 v1.3.0 (Love et al., 2014) and edgeR v.3.32.1 (Robinson et al., 2010) were employed, and both analyses were carried out in RStudio v1.3.1 (RStudio Team, 2020). Prior to differential expression analysis, we removed low count genes using filterByExpr function implemented in edgeR. Principal component analysis (PCA) was performed using the plotPCA function on variance stabilization transformed (vst) count data to assess if the samples of each treatment grouped expectedly. Genes showing |logFC > 1| and adjusted value of *p* <0.05 detected by both algorithms were considered as significantly differentially expressed. Overrepresentation of gene ontology (GO) categories associated with DEG were analyzed using R package topGO (Alexa and Rahnenfuhrer, 2021). The significance was assessed using Fisher’s exact test. GO categories were considered enriched if *p* < 0.01 and were then visualized using ggplot2 v3.3.3 (Wickham, 2016). KEGG enrichment analysis was performed using the R package clusterProfiler v4.2.2 (Wu et al., 2021) and by obtaining *Arabidopsis thaliana* homologs. KEGG terms were considered enriched if the *p*-value was lower than 0.05.

### A/S extraction and quantification

For A/S derivatives quantification, 35 mg lyophilized pulverized roots of each replicate were extracted in 1.5 ml HPLC grade methanol. Briefly, powdered roots were mixed with methanol and placed in an ultrasonic bath (Bandelin Sonorex Digital 10P, Berlin, Germany) for 3 h. The extracts were centrifuged for 10 min at 12,500 rpm, and the supernatant was carefully collected and filtered using 0.22 μm syringe filters. The quantification was performed using HPLC coupled with DAD at a wavelength of 520 nm at the Laboratory of Organic Chemistry, Department of Chemical Engineering, Aristotle University of Thessaloniki, Greece. External calibrations were performed using the following standards: shikonin (Ichimaru, Japan) acetylshikonin (ABCR GmbH Germany), deoxyshikonin (TCI, Belgium), *β,β*–dimethylacrylshikonin (ABCR GmbH Germany), and isovalerylshikonin (TCI, Belgium), of known purity and confirmed by LC–MS identity. Analyses were performed on an ECOM analytical HPLC instrument, model ECS05 (Prague, Czech Republic), utilizing a Fortis SpeedCore C18 column (Cheshire, United Kingdom). The mobile phase consisted of ultrapure water with 0.1% formic acid (A) and acetonitrile (B). Elution was performed using the following solvent gradient: 0 min 30A/70B, 8 min 100B, and 13 min 100B. Prior to the next injection, the column was equilibrated for 5 min with the initial solvent composition. The column temperature was kept at 35 °C. The obtained data were processed with Clarity (DataApex, Prague, Czech Republic).

## Results

### R-73072 leads to an increased A/S biosynthesis in *Lithospermum officinale*

**Figure 1:**
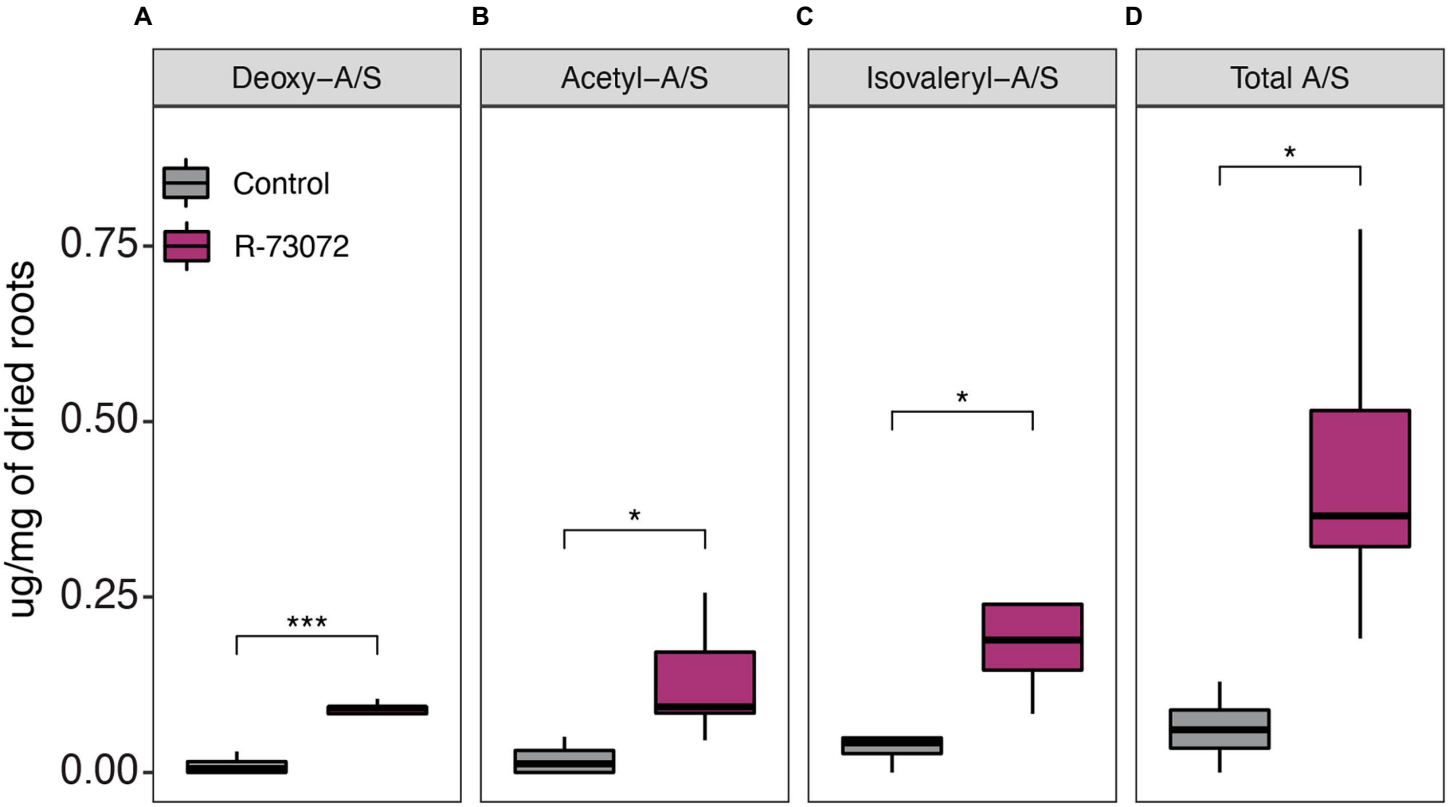
*Chitinophaga* sp. strain R-73072 altered root chemical profiles of *Lithospermum officinale*. R-73072 was point inoculated into growth medium and 4 weeks old shoot cuttings were placed in contact with inoculum. Root tissues were harvested at 5 wpi (~2–3 weeks after root emergence) and A/S were quantified using HPLC coupled with DAD. **(A)** Deoxy-A/S. **(B)** Acetyl-A/S. **(C)** Isovaleryl-A/S. **(D)** Total A/S representing the sum of all quantified alkannin/shikonin derivatives. The asterisks indicate statistically significant differences between two treatments (*p* < 0.05) as evaluated using the Student’s *t*-test.

To determine the impact of R-73072 on A/S biosynthesis, the contents of alkannin/shikonin, and its four derivatives (acetyl-A/S, deoxy-A/S, *β,β*–dimethylacryl-A/S and isovaleryl-A/S) were quantified at 5 weeks post-inoculation (5 wpi) in inoculated and non-inoculated roots of *L. officinale*. Bacterial inoculation led to a significant increase in total A/S (Figure 1). In comparison to non-inoculated control plants, total A/S levels were about six times higher (*p* < 0.05) in inoculated plants (Figure 1D). When comparing A/S derivatives individually, isovaleryl-A/S, acetyl-A/S, and deoxy-A/S also accumulated at significantly higher levels (*p* < 0.05) in inoculated roots as compared to non-inoculated ones (Figures 1A–C), while *β,β*–dimethylallyl-A/S and A/S itself were present in trace amounts. Among the quantified derivatives, isovaleryl-A/S was the most abundant in the inoculated plants, followed by acetyl-A/S and deoxy-A/S. Nonetheless, the differences in individual derivative abundance within inoculated plants were not significant (*p* < 0.05).

Using quantitative real-time PCR (qRT-PCR), we further tested if bacterial inoculation led to enhanced expression of selected key genes of the A/S pathway at 5 wpi and if this time point is optimal for mRNA-seq profiling. Toward this goal, we quantified the expression of 3-hydroxy-3-methylglutaryl reductase (HMGR), p-hydroxybenzoate geranyltransferase (LePGT1), and geranylhydroquinone 3-hydroxylase (LeCYP76B101). Consistent with enhanced A/S biosynthesis, expression levels of two key genes (HMGR and LePGT1), as estimated by qRT-PCR, increased significantly (*p* < 0.05) in R-73072 challenged roots as compared to non-inoculated roots, while that of LeCYP76B101 was marginally significant (*p* < 0.1; Supplementary Figure 1).

### R-73072 reprograms the root transcriptome of *Lithospermum officinale*

To gain insight into the mechanism of enhanced A/S biosynthesis and to study global root transcriptional changes in response to *Chitinophaga* sp. strain R-73072, we next performed a comparative transcriptomic analysis of quadruplicate bacterial challenged and non-challenged roots of *L. officinale* at 5 wpi. Overall, mRNA sequencing yielded 265 million paired-end reads (average 33.18 million reads per sample), ~90% of them passed the quality control. On average, 90% (~26 million reads per sample) of the filtered reads mapped back to the *L. erythrorhizon* reference genome (Auber et al., 2020) where a majority (83%) mapped uniquely to the exonic regions (85%; Supplementary Data S1).

**Figure 2:**
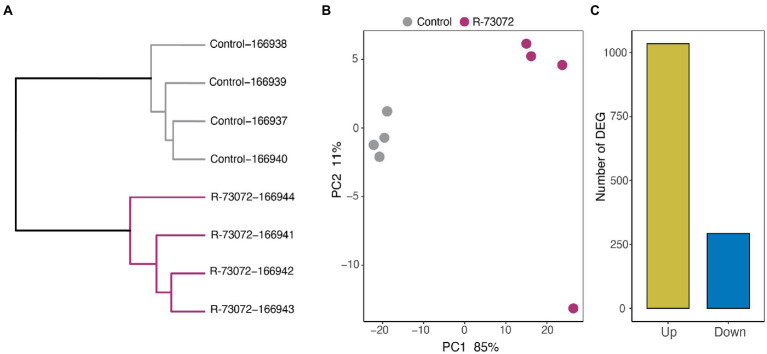
*Chitinophaga* sp. strain R-73072 reprogramed root transcriptional profiles of *L. officinale*. **(A)** Clustering of root mRNA-seq profiles of *L. officinale*. Samples were clustered based on Euclidian distances of variances stabilized gene expression profiles as implemented in DESeq2. **(B)** Principal component analysis (PCA) of challenged and non-challenged root mRNA-seq profiles. **(C)** Numbers of genes significantly differentially expressed (|log_2_FC| > 1 and FDR < 0.05) in roots of *L. officinale* in response to inoculation with R-73072 as compared to controls.

Hierarchical clustering of *L. officinale* root transcriptomes clustered the treatments into two distinct groups: one containing all four samples of plants challenged by the R-73072 while the second comprising samples of non-inoculated plants (Figure 2A). Similarly, principal component analysis (PCA) grouped all samples into two distinct clusters reflecting the respective treatments (Figure 2B). To gain an overall insight into R-73072 modulated *L. officinale* transcriptomes, differentially expressed genes (DEG) were identified. R-73072 inoculation resulted in 1,328 DEG (FDR < 0.05 and |log2FC| > 1), the majority of which were upregulated in response to bacterial inoculation (Figure 2C; Supplementary Data S2–S4). Taken together, these results showed the effectiveness of R-73072 in the modulation of root transcriptional profiles of *L. officinale.*

### R-73072 regulates processes associated with plant defense and secondary metabolites biosynthesis

**Figure 3:**
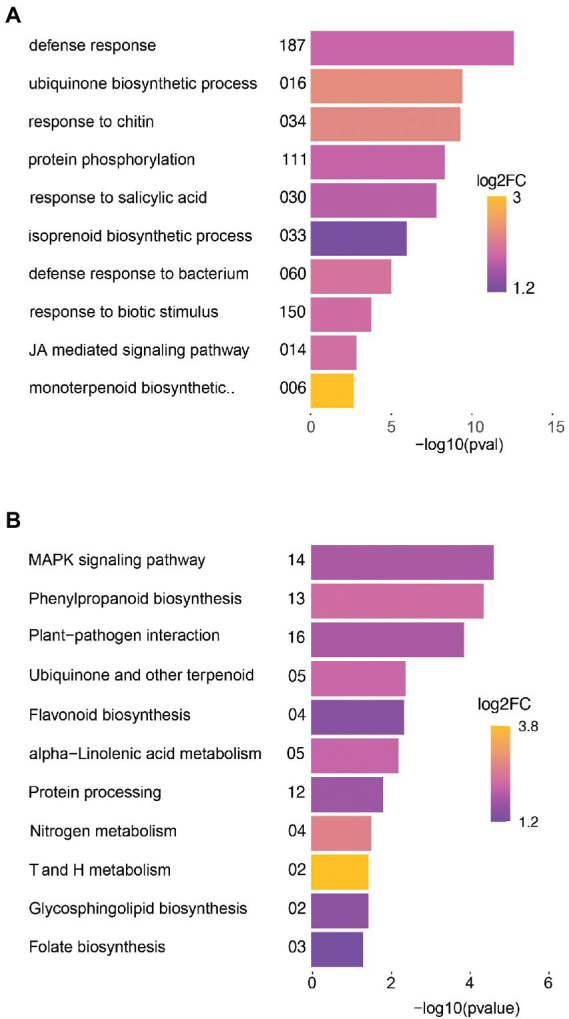
Gene Ontology (GO) and pathway (KEGG) enrichment analysis of genes upregulated in response to *Chitinophaga* sp. strain R-73072 as compared to control plants. **(A)** GO enrichments of biological processes. **(B)** KEGG pathways. The length of bars represents the statistical significance of terms as estimated by Fisher’s exact test. GO or KEGG terms with *p*-value < 0.01 or < 0.05, respectively, were considered enriched. Numbers on the left of horizontal bars depict the number of differentially expressed genes in the corresponding GO or KEGG term. The variation in colors of each horizontal bar reflects the average log_2_FC in the respective GO or KEGG term. Only the most interesting terms are shown here, while a complete list of significant GO or KEGG terms is presented in Supplementary Data S5–S10.

To understand the biological relevance of the R-73072 responsive root transcriptome of *L. officinale*, the identified DEG were subjected to Gene Ontology (GO) and Kyoto Encyclopedia of Genes and Genomes (KEGG) enrichment analysis. In the biological process category (BP) of GO terms, a majority of genes were significantly enriched (*p* < 0.01) with terms associated with “defense response,” “defense response to bacterium,” “response to biotic stimulus,” and activation of the phytohormonal signaling pathways (Supplementary Data S5–S7). In the molecular function (MF) group, GO terms associated with kinase, transferase, and heme-binding activity were significantly overrepresented (*p* < 0.01; Supplementary Data S5–S7). When performing GO enrichments separately for up-and downregulated genes, the former had a strong signature of plant defense (Figure 3A). In agreement with GO enrichments, KEGG overrepresentation analysis of genes induced in R-73072 treated plants also revealed an enrichment of DEG associated with plant defense (*p* < 0.05; Figure 3B; Supplementary Data S8). Apart from the activation of plant defense by R-73072, we observed the enrichment of upregulated genes associated with the SMs biosynthesis such as “ubiquinone biosynthetic process,” “isoprenoid biosynthetic process,” and “monoterpenoid biosynthetic process” (Figure 3A). Similarly, at the pathway level, genes involved in “phenylpropanoid biosynthesis, and ubiquinone” and “other terpenoid-quinone biosynthesis” were enriched among DEG (Figure 3B). Overrepresentation of genes associated with the aforementioned KEGG or GO terms is noteworthy because they encompass genes that are directly connected to A/S production in *L. officinale* and other Boraginaceae (Suttiyut et al., 2022).

### R-73072 alters the expression of genes possibly involved in plant defense

A higher number of DEG associated with plant defense suggests that the presence of R-73072 was perceived by the plant. Receptor-like kinases (RLKs) and-proteins (RLPs) are generally involved in plant defense and recognition of microbe-pathogen-associated molecular patterns (M/PAMP) to modulate downstream signaling cascades (Yu et al., 2017). In line with this, the expression of several genes encoding different members of RLKs such as LysM domain-containing-RLK (LYKs) and cysteine-rich receptor-like kinases (CRK), among others were modulated in R-73072 inoculated plants as compared to non-challenged plants (Table 1; Supplementary Data S3). Importantly, homologs of several of these genes in *Arabidopsis thaliana* and other model plant species have been shown to play critical functions in plant immunity (Yu et al., 2017). For instance, two genes encoding an LYK4 protein showed enhanced expression in response to R-73072 inoculation (Table 1). In *Arabidopsis thaliana*, the *lyk4* mutant is more susceptible to both bacterial and fungal pathogens (Wan et al., 2012). Furthermore, three genes encoding CRK (CRK2, CRK10, and CRK25) modulated their expression in response to R-73072 inoculation (Table 1; Supplementary Data S3). CRK2 is involved in PAMP-triggered reactive oxygen species production, and callose deposition in roots and is required for resistance against *Pseudomonas syringae* pv. tomato DC3000 (Kimura et al., 2020). In addition to LYK4 and CRKs, we observed an upregulation of PBL2, PBL18, and PBL19 in R-73072 inoculated plants (Table 1; Supplementary Data S3). PBLs are receptor-like cytoplasmic kinases and expression levels of its members such as PBL2 have been shown to strongly upregulate upon inoculation with different elicitors or living bacterium and contribute to PAMP-triggered signaling downstream of well-characterized receptor FLS2 (Zhang et al., 2010; Wang et al., 2015). Apart from modulation of the aforementioned RLKs by R-73072, inoculation by this bacterial strain also enhanced the accumulation of transcripts of pre-PAMP-induced secreted peptide 1 (prePIP1) and its receptor RLK7 (Table 1). prePIP1 encodes an endogenous peptide PIP1 that is secreted into the apoplast and is recognized by RLK7 to amplify the immune responses (Hou et al., 2014). The increased expression of RLKs on one hand could indicate the abundance of pattern recognition receptors at the cell surface. On the other hand, the upregulation of prePIP1 might suggest increased availability of endogenous secreted peptides which together with MAMP of R-73072 origin might have led to amplification of defense responses at the transcriptional level in inoculated plants.

**Table 1:** Selected differentially expressed plant immunity and defense-related genes responding to *Chitinophaga* sp. strain R-73072 inoculation in *Lithospermum officinale.*

Gene	Name	Homolog	log_2_FC**	FDR**
**Receptor-like kinases/proteins (RLKs/RLPs)**				
Leryth_008018	SOBIR1	At2g31880	1.63/1.60	3.24E-05/2.92E-31
Leryth_009940	SOBIR1	At2g31880	1.33/1.31	3.37E-05/4.79E-31
Leryth_008055	CRK2	At1g70520	1.21/1.19	5.29E-05/9.44E-27
Leryth_014565	LYK4	At2g23770	1.09/1.07	8.51E-05/ 6.25E-21
Leryth_015096	LYK4	At2g23770	2.46/2.42	5.58E-05/ 3.68E-27
Leryth_014483	LYK5	At2g33580	1.14/1.12	4.01E-05/3.68E-27
Leryth_023675	LYK5	At2g33580	3.59/3.48	0.00053/1.76E-15
Leryth_014150	RLK7	At1g09970	4.51/4.29	0.0018/ 2.11E-10
Leryth_004923	PBL2	At1g14370	3.73/3.66	0.00084/3.45E-20
Leryth_005473	PBL19	At5g47070	1.33/1.27	0.001/1.78E-09
Leryth_015108	RLCK176	Os05g0110900	1.56/1.55	1.95E-05/1.10E-50
**Defense-related genes**				
Leryth_006253	prePIP1	At4g28460	5.07/4.67	0.009/6.49E-07
Leryth_019906	PR1		1.24/1.16	0.003/ 1.85E-06
Leryth_021873	PR1		−2.34/−2.16	0.002/ 1.57E-05
Leryth_010259	FMO1	At1g19250	3.46/3.07	0.016/0.00019
Leryth_021258	CHI5	At3g54420	5.41/5.36	0.00014/2.16E-31
Leryth_021259	CHI5	At3g54420	3.89/3.87	3.24E-05/9.73E-65
**SA metabolism and signaling**				
Leryth_006114	SARD1	At1g73805	3.72/3.68	8.15E-05/5.58E-48
Leryth_009394	SARD1	At1g73805	3.42/3.36	0.0002/3.03E-28
Leryth_003919	WRKY70	Solyc03g095770	3.78/3.62	0.0014/3.47E-11
Leryth_007513	WRKY70	Solyc03g095770	3.39/3.33	0.0002/5.17E-26
Leryth_011094	WRKY70	Solyc03g095770	3.36/3.30	0.0004/1.13E-24
Leryth_024007	WRKY70	Solyc03g095770	2.42/2.37	0.0001/9.78E-32

^*^log_2_FC, log_2_fold change, values above and below are from edgeR and DEseq2, respectively.

^**^FDR, false discovery rate, values above and below are from edgeR and DEseq2, respectively.

### R-73072 inoculated plants are more resistant to subsequent infection by the leaf fungal pathogen *Botrytis cinerea*

**Figure 4:**
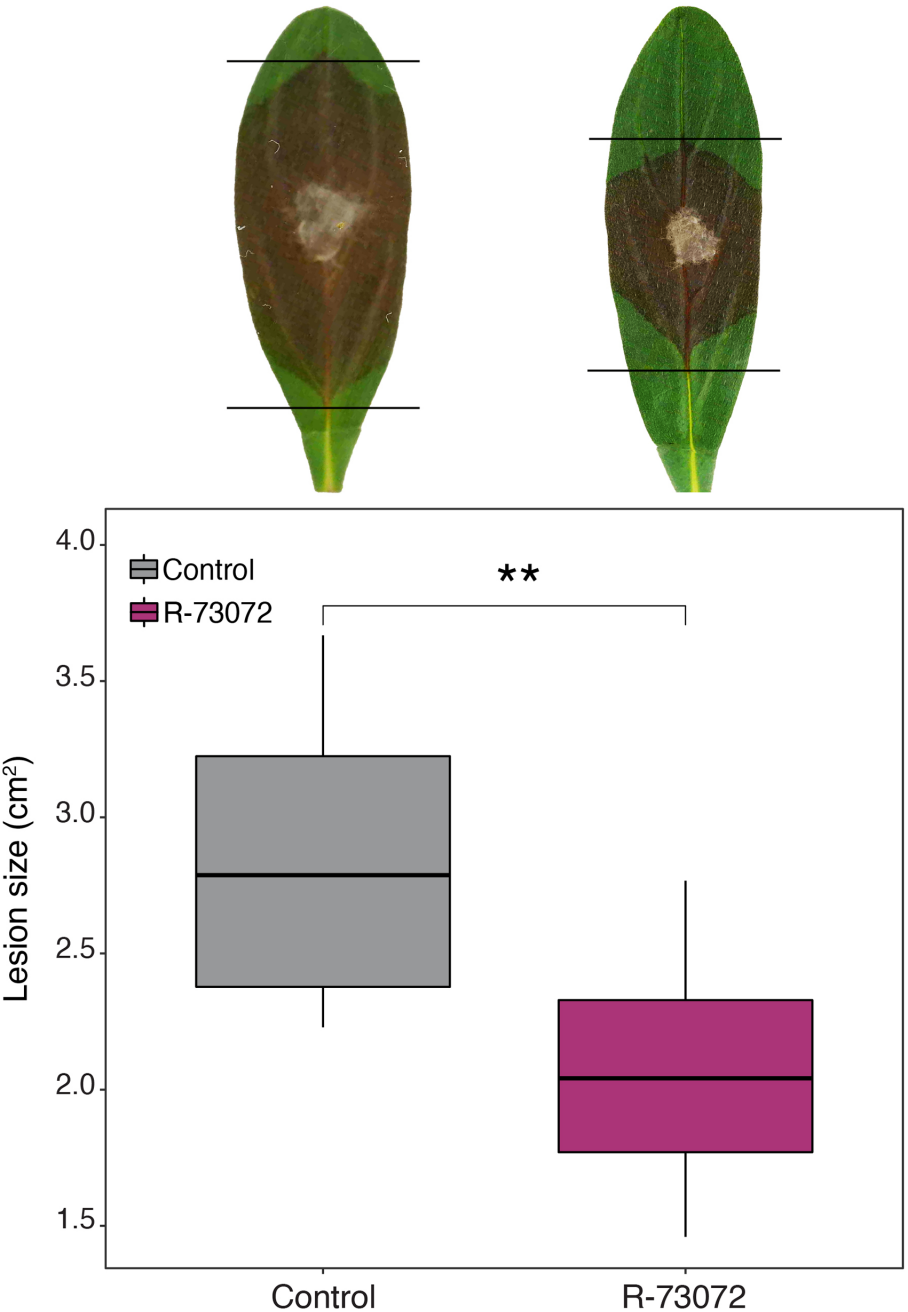
*Chitinophaga* sp. strain R-73072 reduced leaf infection of *Botrytis cinerea* in *L. officinale*. Plants were root inoculated twice with R-73072 and grown in the greenhouse. After that, leaves of inoculated and non-inoculated plants were detached, placed on water-agar in Petri plates, and infected with equal-sized mycelial plugs of *B. cinerea.* The asterisks indicate statistically significant differences in lesion size of detached leaves between the two treatments (*p* < 0.01, *n* = 10).

Higher induction of defense-related genes prompted us to investigate if R-73072 could enhance resistance against pathogens in *L. officinale*. To test this hypothesis, roots of *L. officinale* plants were twice-inoculated with R-73072, leaves were removed 2 weeks after second inoculation, and challenged with the fungal pathogen *B. cinerea.* Root inoculation of *L. officinale* with R-73072 led to enhanced protection of leaves against *B. cinerea* with a significant (*p* = 0.002) reduction in leaf lesion size in bacterized plants as compared to non-bacterized plants (Figure 4). This suggests that R-73072 possesses the ability to trigger resistance and to provide extended protection against *B. cinerea* in *L. officinale*.

### R-73072 simultaneously activates ET and JA biosynthesis and signaling genes

**Figure 5:**
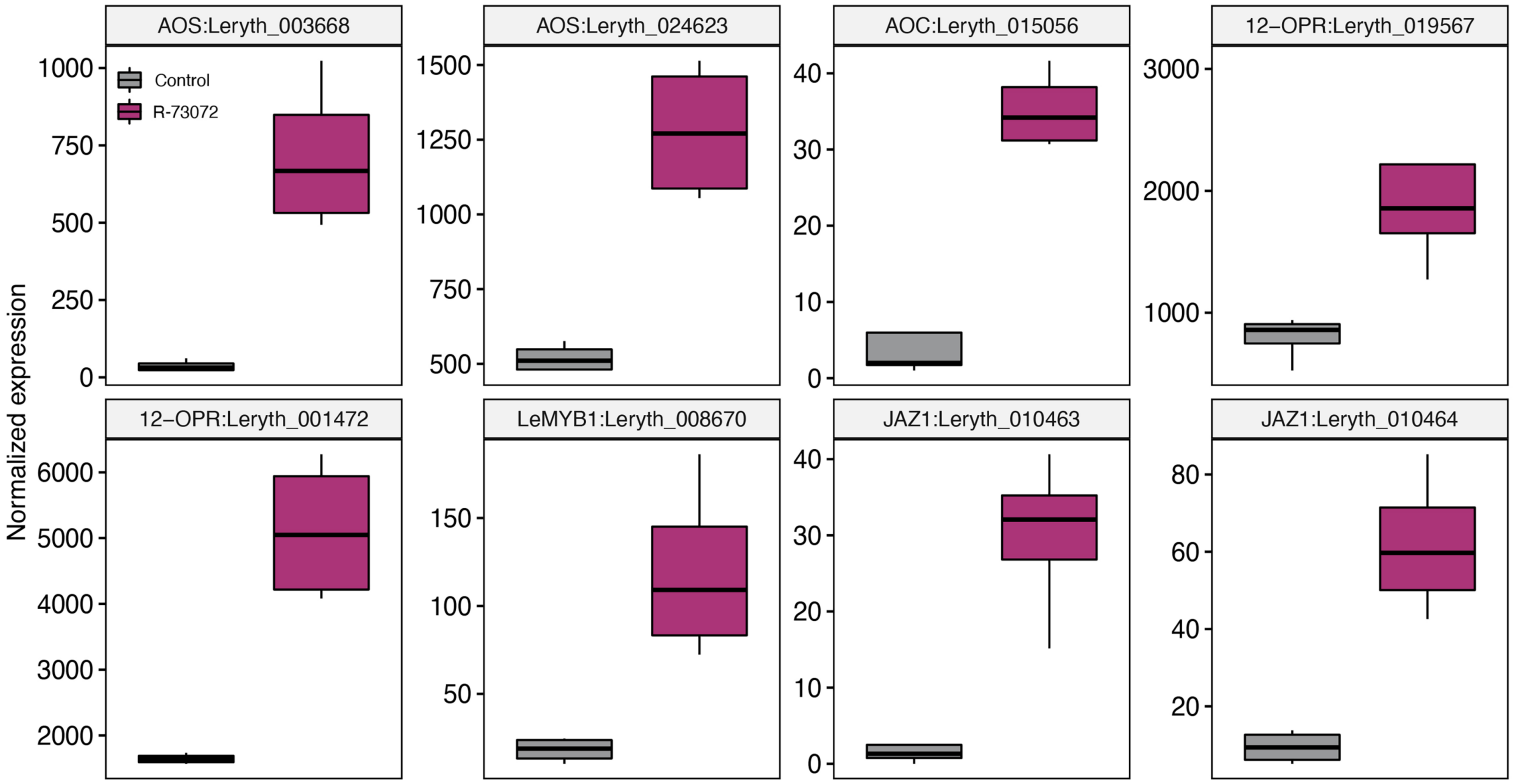
*Chitinophaga* sp. strain R-73072 activated jasmonate (JA) biosynthesis and signaling genes in *L. officinale.* Boxplots represent normalized counts of different JA biosynthetic and signaling genes in challenged and non-challenged plants. *n* = 4 replicates for each treatment. All genes depicted here were significant at FDR < 0.05 and showed at least |log_2_FC| > 1 in R-73072 inoculated plants as compared to control plants. AOS, allene oxide synthase; AOC, allene oxide cyclase; 12-OPR, 12-oxophytodienoate reductase; JAZ, jasmonate ZIM-DOMAIN protein.

**Figure 6:**
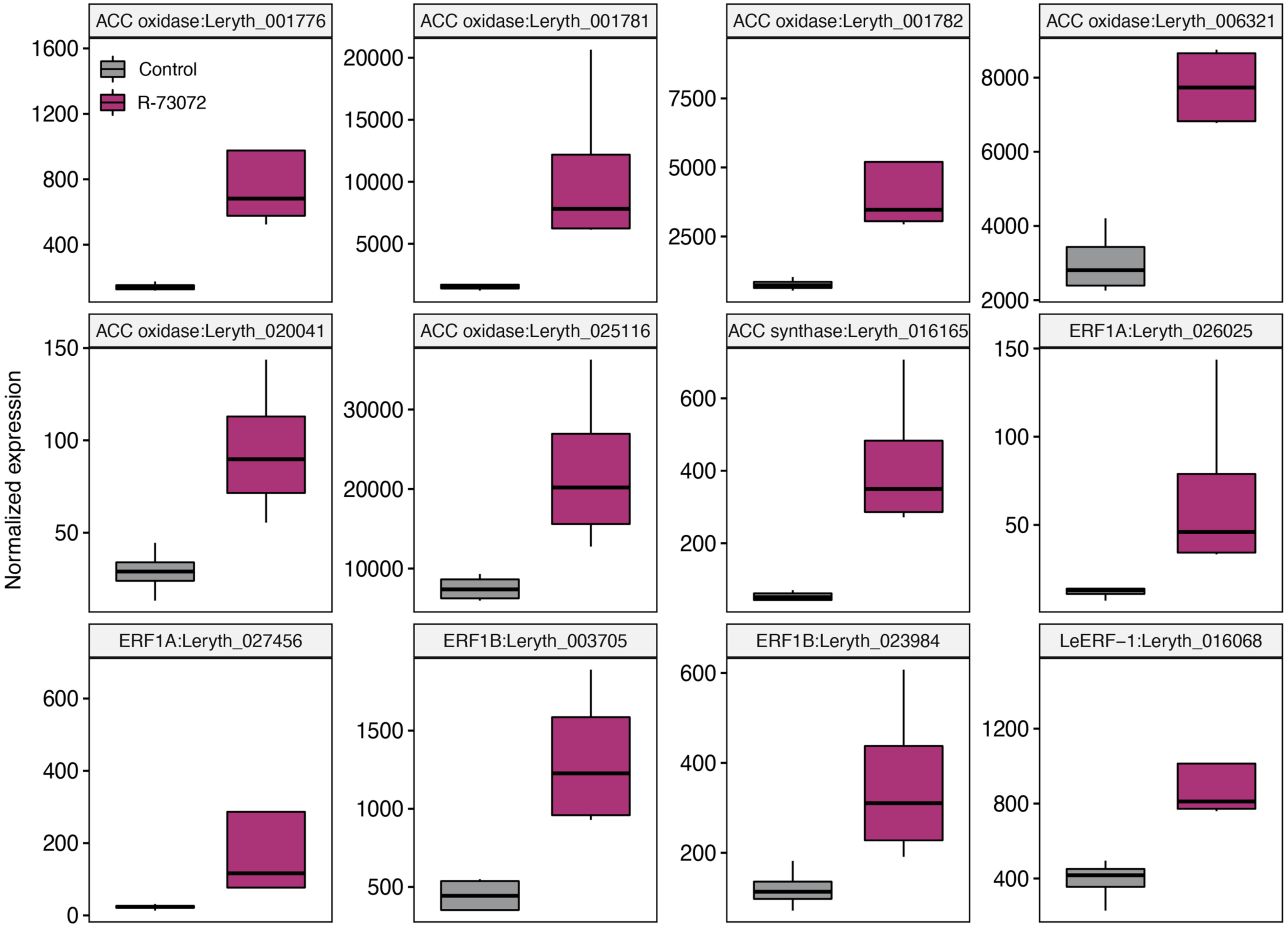
*Chitinophaga* sp. strain R-73072 activated ethylene (ET) biosynthesis and signaling genes in *L. officinale.* Boxplots depict normalized counts of different ET biosynthetic and signaling genes in challenged and non-challenged plants. *n* = 4 replicates for each treatment. All genes depicted showed significance at FDR < 0.05 and at least |log_2_FC| > 1 in R-73072 inoculated plants as compared to control plants. ACC oxidase, 1-aminocyclopropane-1-carboxylic acid oxidase; ACC synthase, 1-aminocyclopropane-1-carboxylic acid synthase; ERF1A/B, ethylene response factor 1A/1B.

Several genes that participate in defense-related phytohormone metabolism and signaling were induced by R-73072 (Supplementary Data S6). These genes encode enzymes for JA (LOX, AOS, AOC, and 12-OPR; Figure 5) and ET (ACC synthase and ACC oxidase; Figure 6) biosynthesis while others have been implicated in JA (LeMYB1 and JAZ1; Figure 5) and ET (LeERF1-like and ERF1; Figure 6) signaling. Though MYC2, a master regulator of JA-mediated transcriptional responses (Liu et al., 2019), was not among the identified DEG, its expression was marginally significant in R-73072 inoculated plants (log2FC = 0.99 and FDR < 0.05). Apart from JA and ET biosynthetic and signaling genes, we observed that a few of the SA-responsive genes were also differentially modulated by R-73072. Among them were PR-like genes (PR1 and PR2), SARD1, and WRKY70 (Table 1; Supplementary Data S3). PR-1 is generally considered a marker gene of SA-mediated resistance, while SARD1 and WRKY70 are transcription factors that regulate SA metabolism and SA-coordinated signaling, respectively (Peng et al., 2021).

### R-73072 modulates the expression of genes involved in A/S biosynthesis

**Figure 7:**
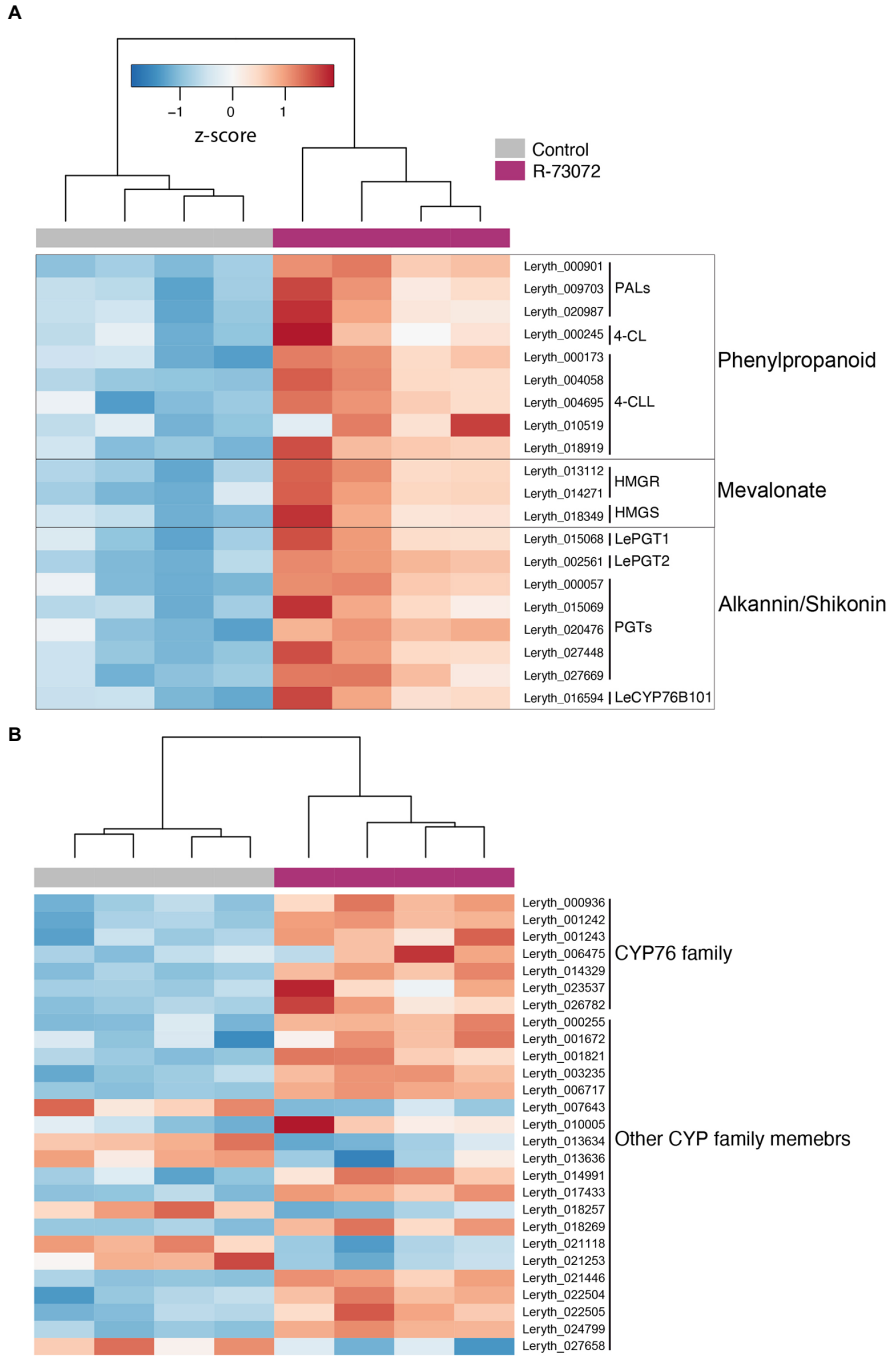
*Chitinophaga* sp. strain R-73072 induced the expression of key genes of the precursors and A/S pathways to upregulate A/S biosynthesis in *L. officinale.* **(A)** Heatmap of variance stabilized expression profiles of key genes of the precursor phenylpropanoid, and mevalonate and A/S pathways. **(B)** Gene expression of CYP76 and other CYP family members in challenged and non-challenged roots of *L. officinale*. All genes depicted here are significant at FDR < 0.05 and show at least |log_2_FC| > 1 in R-73072 inoculated plants as compared to control plants. PAL, phenylalanine ammonia lyase; 4-CL, 4-coumarate ligase; 4-CLL, 4-coumarate ligase-like; HMGR, 3-hydroxy-3-methylglutaryl-CoA reductase; HMGS, 3-hydroxy-3-methylglutaryl-CoA synthase; PGT, 4-hydroxybenzoate-3-geranyltransferase; CYP, cytochrome P450.

To investigate the effect of R-73072 inoculation on A/S biosynthesis, we examined the expression level of genes associated with A/S production. As apparent from GO and KEGG enrichment analysis, R-73072 modulated the expression levels of genes related to A/S metabolism (Figure 7). These included genes of the precursors’ phenylpropanoid and mevalonate route as well as those that encode enzymes of the downstream core A/S pathway. Of the phenylpropanoid pathway, expression levels of genes encoding phenylalanine ammonia-lyase, 4-coumarate-CoA ligase, and 4-coumarate-CoA ligase-like were increased in response to R-73072. In an earlier work (Ahmad et al., 2022), using comprehensive co-expression network analysis, we identified that one of the genes (Leryth_018919) encoding 4-coumarate-CoA ligase-like protein might be involved in 4-HBA production, one of the two precursors required for A/S biosynthesis in Boraginaceae. In the present work, the same gene showed a 25-fold increase in expression upon R-73072 inoculation (Figure 7A), further reinforcing its potential importance in 4-HBA biosynthesis. Of the mevalonate pathway, bacterial inoculation induced the expression of gene encoding hydroxy-methylglutaryl-coenzyme A reductase and hydroxymethylglutaryl-CoA synthase. In congruence with enhanced responsiveness of genes involved in the biosynthesis of precursors, genes of the core A/S pathway such as p-hydroxybenzoate-geranyltransferase (LePGT1 and LePGT2, among other copies) and a cytochrome P450 (LeCYP76B101) showed a significant increase in expression in R-73072 inoculated roots of *L. officinale.* In addition to the characterized LeCYP76B101, seven other CYP76-like genes with unknown functions were expressed at significantly higher levels in R-73072 challenged roots (Figure 7B). This is noteworthy since CYP76-like genes have been suggested to catalyze missing steps in A/S biosynthesis and thus could be important functional targets (Song et al., 2020).

## Discussion

Plants harbor a wide range of microorganisms and their interaction with plants can significantly influence the plant transcriptional machinery. Furthermore, specific microbial partners can trigger the biosynthesis of SMs that not only confer enhanced fitness to plants but are also commercially valuable. In a recent work, Rat et al. (2021) showed that *Chitinophaga* sp. strain R-73072 promoted the biosynthesis of A/S, defense-related SMs of higher pharmaceutical importance. However, the mechanisms by which R-73072 increases the A/S production remain not understood to date. Thus, we investigated the transcriptional mechanisms behind the enhanced biosynthesis of A/S in the interaction between *L. officinale* and *Chitinophaga* sp. strain R-73072 *via* comparative transcriptomics of roots challenged with this bacterial strain.

Our data showed that R-73072 inoculated to the roots of *L. officinale* resulted in a significant reprogramming of the root transcriptome, altering the expression of 1,329 genes. Transcriptome data further indicated that the presence of R-73072 was recognized by the plant leading to changes in the expression of genes that are typically involved in plant immunity (e.g., RLK) when plants are exposed to phytopathogens (Yu et al., 2017). However, R-73072 appeared non-pathogenic to *L. officinale* as we did not observe any visible symptoms or growth inhibition (Supplementary Figure 2). These findings are in accordance with earlier observations where no growth defects nor a loss in biomass were found during greenhouse and *in vitro* investigations (Varela-Alonso et al., 2022). Instead, defense-related transcriptional responses upon R-73072 interaction might confer protection against further potentially unwanted microorganisms. Induction of RLK and RLP, together with a plethora of defense-related genes, has been observed in response to *Pseudomonas fluorescens* SS101 and *Sphingomonas melonis* Fr1 in *Arabidopsis thaliana* (van de Mortel et al., 2012; Vogel et al., 2016). Both bacteria are known to provide protection against *Pseudomonas syringae* DC3000 (van de Mortel et al., 2012; Vogel et al., 2016). Intriguingly, plant protective activities of different *Chitinophaga* spp. have been recently demonstrated in sugar beet against phytopathogenic fungi (Carrión et al., 2019). It was suggested that protection might operate through increased secretion of chitinases by *Chitinophaga* spp. and/or local and systemic resistance in inoculated plants (Carrión et al., 2019). Our findings of an elevated expression of several RLK and RLP genes in inoculated plants thus might point toward potential kinases that could be involved in this specific plant-microorganism interaction and might have initiated the signaling cascades to activate the expression of observed local downstream defense-related genes (e.g., endochitinases, peroxidases, phenylalanine ammonia-lyase, and secondary metabolite related genes). This all can be supported by our findings, where R-73072 was able to reduce an infection by *Botrytis cinerea* in *L. officinale*. A similar mode of action has been described for beneficial microorganisms acting as an antifungal agent against *B. cinerea* and other phytopathogens in several plant species (Chen et al., 2020; Li et al., 2020), pointing toward the possibility of R-73072 as a biocontrol agent. Although the direct evidence and mechanism of R-73072 mediated protection remain to be investigated, our data provide the first insight regarding the potential involvement of R-73072 in activating the plant defense machinery and protection against fungal infection in *L. officinale.*

Besides its potential to induce the plant defense machinery and provide protection against a fungal pathogen, R-73072 also interfered with JA and ET signaling. The two phytohormones (JA and ET) are well-known defense signaling compounds but also induce the A/S biosynthesis pathway in different Boraginaceae species (Yazaki et al., 1997b; Hao et al., 2014; Fang et al., 2016a,b; Ahmad et al., 2022). In the earlier (Varela-Alonso et al., 2022) and present study, R-73072 clearly enhanced the biosynthesis of A/S in *L. officinale*, which can be explained—besides the obvious phenotypic response—by the widespread upregulation of genes involved in A/S biosynthesis and associated transcription factors (Zhang et al., 2011; Zhao et al., 2014, 2015). LeMYB1 was the first characterized JA-responsive transcription factor that positively regulates A/S metabolism in *L. erythrorhizon* (Zhao et al., 2014, 2015). We showed that the expression level of this regulatory gene together with ET and JA biosynthesis genes was higher in R-73072 inoculated plants. In addition, transcript levels of 12 additional ET-responsive transcription factors including ERF1 and LeERF1 were increased in response to R-73072 inoculation (Supplementary Data S3). ERF1 has been shown to integrate signals from JA and ET pathways in *Arabidopsis thaliana* (Lorenzo et al., 2003). Furthermore, LeERF-1 has been suggested to positively regulate A/S production (Zhang et al., 2011; Fang et al., 2016a). Altogether, these results suggest that R-73072 activates JA and ET biosynthesis and signaling in *L. officinale.* As both phytohormones have been shown to enhance A/S biosynthesis in different Boraginaceae species (Yazaki, 2017), it could be possible that R-73072 increases the endogenous levels of these phytohormones which, in turn, activates the expression of LeMYB1/LeERF-1 to induce transcription of genes that encode enzymes of the A/S pathway leading to an enhanced A/S biosynthesis in *L. officinale*. Furthermore, higher levels of A/S production and co-regulation of genes involved in plant defense in R-73072 inoculated roots together with demonstrated antimicrobial activities of A/S (Brigham et al., 1999) strongly suggest that A/S might be an important component of the plant defense against pathogens in Boraginaceae.

To the best of our knowledge, the current study is the first report showing that R-73072 triggers substantial defense responses in *L. officinale*. As a consequence, an enhanced systemic protection of *L. officinale* against the tested fungal pathogen was observed. Furthermore, we showed for the first time that JA and ET-signaling might be involved in the R-73072-mediated enhancement of the A/S biosynthesis in *L. officinale.* However, in future studies, it would be crucial to further investigate the role of these phytohormones in the R-73072-*L. officinale* interaction in context of A/S biosynthesis and to understand the mechanism by which R-73072 provides systemic protection against *B. cinerea.*

## Data availability statement

The data presented in the study are deposited in the NCBI Sequence Read Archive (https://www.ncbi.nlm.nih.gov/sra) repository, accession number PRJNA854093.

## Author contributions

MA, EM, and AA planned and designed the research. MA performed the *in vitro* experiment and laboratory work. MA did bioinformatics, statistical analysis, and visualization, supported by EM. AK and AA performed metabolite analysis. AA prepared the *in vitro* plants and performed the greenhouse and detached leaf assay experiment. MA prepared the manuscript with input from all co-authors. All authors contributed to the article and approved the submitted version.

## Funding

The research was supported by the European Union’s Horizon 2020 research and innovation program under the Marie Skłodowska-Curie grant agreement No. 721635, MICROMETABOLITE ITN project.

## Acknowledgments

We would like to acknowledge Angélique Rat and Anne Willems for providing *Chitinophaga* sp. strain R-73072 culture, Angela Sessitsch for the coordination of the ITN project, and Jennifer H. Wisecaver for providing the assembled genome of *Lithospermum erythrorhizon*. We are grateful to Ovidiu Paun and Angela Sessitsch for critically reviewing the manuscript.

## Conflict of interest

MA and EM were employed by AIT Austrian Institute of Technology GmbH. AA and CS were employed by Institut für Pflanzenkultur GmbH & Co. KG.

The remaining authors declare that the research was conducted in the absence of any commercial or financial relationships that could be construed as a potential conflict of interest.

## Publisher’s note

All claims expressed in this article are solely those of the authors and do not necessarily represent those of their affiliated organizations, or those of the publisher, the editors and the reviewers. Any product that may be evaluated in this article, or claim that may be made by its manufacturer, is not guaranteed or endorsed by the publisher.

## Supplementary material

The Supplementary material for this article can be found online at: https://www.frontiersin.org/articles/10.3389/fmicb.2022.978021/full#supplementary-material
