# Supplementary material for: Transcriptional dynamics of Chitinophaga sp. strain R-73072-mediated alkannin/shikonin biosynthesis in Lithospermum officinale
Source: Front Microbiol. 2022 Aug 22;13:978021. doi: 10.3389/fmicb.2022.978021 (PMC9441710; doi:10.3389/fmicb.2022.978021)
Supplement: Supplementary file 2 [file Image_1.pdf]

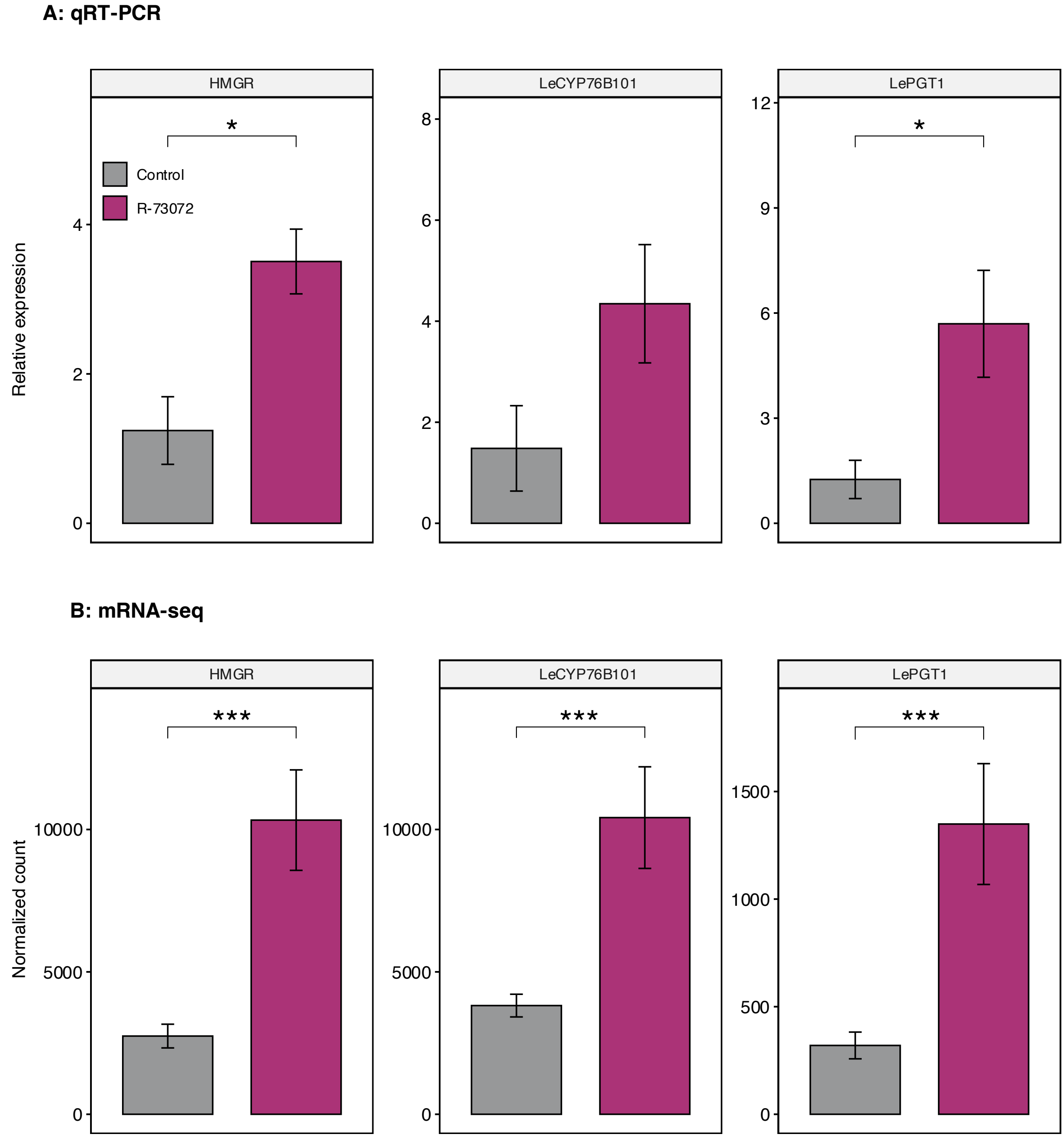

Supplementary Figure 1: *Chitinophaga* sp. strain R-73072 induced the expression of key genes of the precursors and A/S pathways to upregulate A/S biosynthesis in *L. officinale*. Expression of key genes of alkannin/shikonin pathway measured by **A)** qRT-PCR **B)** mRNA-seq. For qRT-PCR, LeACT7 was used as a reference. Values are means ± se of four replicates. Each replicate is a pool of roots from three individual plants grown in the same glass jar. The asterisks indicate statistically significant differences between the two treatments (p or FDR < 0.05).
