# Supplementary material for: Transcriptional dynamics of Chitinophaga sp. strain R-73072-mediated alkannin/shikonin biosynthesis in Lithospermum officinale
Source: Front Microbiol. 2022 Aug 22;13:978021. doi: 10.3389/fmicb.2022.978021 (PMC9441710; doi:10.3389/fmicb.2022.978021)
Supplement: Supplementary file 3 [file Image_2.pdf]

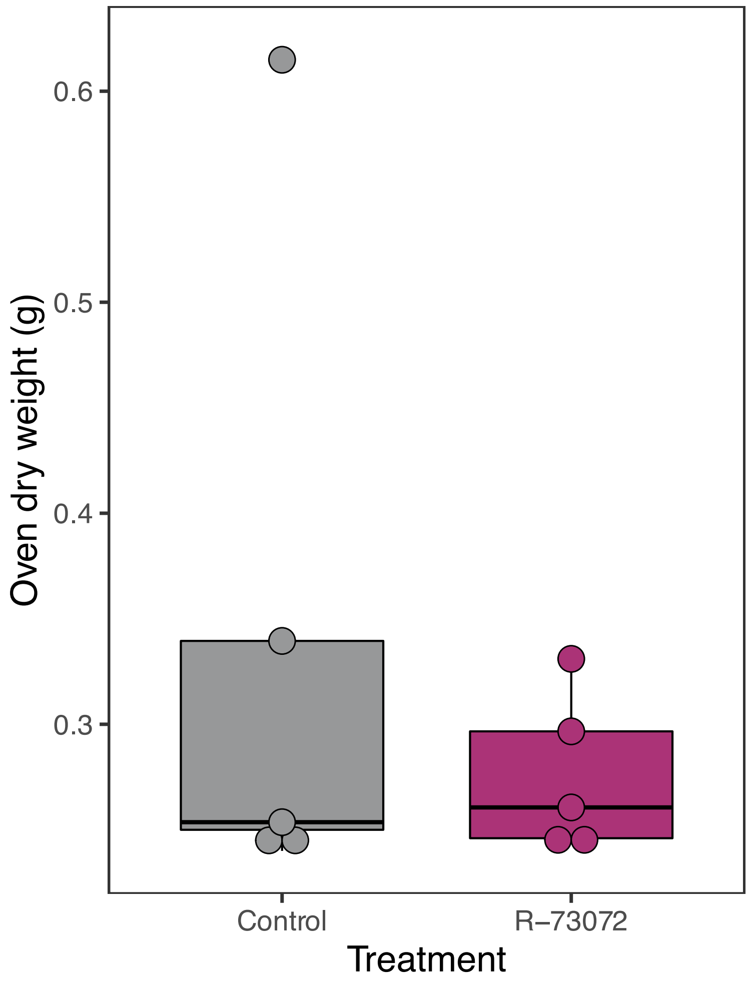

Supplementary Figure 2: Effect of *Chitinophaga* sp. strain R-73072 on plant growth. Boxplot represents distribution of shoot oven dry weight of *L. officinale* in R-73072 inoculated and control plants. n = 5 replicates for each treatment. Each replicate is a pool of shoots from three individual plants grown in the same glass jar. The distributions are non-significant (p = 0.42) as evaluated by t-test.
